# Supplementary material for: Expression of the ripAA Gene in the Soilborne Pseudomonas mosselii Can Promote the Control Efficacy against Tobacco Bacterial Wilt
Source: Biology (Basel). 2022 Aug 4;11(8):1170. doi: 10.3390/biology11081170 (PMC9405386; doi:10.3390/biology11081170)
Supplement: Supplementary file 1 [file biology-11-01170-s001.zip › biology-1816586-supplementary.pdf]

Biology

Article

Expression of *ripAA* gene in a soil-born *Pseudomonas mosselii* can promote the control efficacy against tobacco bacterial wilt

Tao Zhuo †, Shiting Chen †, Dandan Wang, Xiaojing Fan, Xiaofeng Zhang * and Huasong Zou *

1 State Key Laboratory of Ecological Pest Control for Fujian and Taiwan Crops, College of Plant Protec-tion, Fujian Agriculture and Forestry University, Fuzhou, China

* Correspondence: zhangxiaofeng911@163.com, huasongzou@163.com.

† These authors contributed equally to this work

Table S1. Primers used for molecular cloning and RT-PCR analysis in this study

Primer pair	Sequence(5′-3′)	Cutting sites	Description or purpose
**Primers used for molecular cloning**			
Hy1.F / Hy1.R	TCGGATCCCTACGAGCTGAGCA<br>TCGTCGACCTGAATTCAGGAAGTAGTAG<br>TTGCCGTCGTA	*Bam*HI/*Sal*I	A 604-bp DNA fragment containing 5′ terminus of a hypothetical gene in *Pseudomonas mosselli* A1

Hy2.F / Hy2.R	TCGAATTCTCAAGCTTCCAACAACTGCGCGGTG<br>TCGTCGACCTGCTTGATATTGGCTTCC	*Eco*RI/*Sal*I	A 677-bp DNA fragment of 3′ terminus of a hypothetical gene in *Pseudomonas mosselli* A1
RipAA.F / RipAA.R	TCGAATTCGCTCGGGCCAAAACGCTT<br>TCAAGCTTACAAGTCCTCTTCAGAAATGAGCTTTTGCTCGCCGTCGCTATCGCTATCG	*Eco*RI/*Hin*dIII	A 1100-bp DNA fragment containing *RipAA* gene and its native promoter
PRipAA.F/PRipAA.R	ACGGTACCCGCACGAGGAT<br>TGCTCGAGCTCAATCTCCTGAGTTTCAAATAG	*Kpn*I/*Xho*I	A 276-bp *RipAA* gene promoter cloned to fuse with *lacZ*
**Primers used for RT-PCR analysis**			
16S rRNA.F/ 16S rRNA.R	GTAAAGCGCGCGTAGGTGGT<br>CGAAGGCACCAATCCATCTCT		464-bp
RipAA.F/ RipAA.R	TGACGACGGGAGGGTGACATAT<br>CATGCACGCAGTTGCTGTATT		366-bp
Nt-EF1α-F/ Nb-EF1α-R	TGAGATGCACCACGAAGCTC<br>CCAACATTGTCACCAGGAAGTG		51-bp [1]
qNt1-F/ qNt1-R	TGACAGCCTTTCTGCACTTGAT<br>CTGAGAATCCGTAGAACCACCA		133-bp, detection for gene Nitab4.5_0011671g0010.1
qNt2-F/ qNt2-R	TAGATCAGGCTAAACAAAGGAT<br>TAGTCGCACTGGGTATGGTATT		128-bp, detection for gene Nitab4.5_0000273g0080.1
qNt3-F/ qNt3-R	AATCTGACAGCAGGAAGGAATC<br>TGTACGTGGTTAGTCACTGGAT		119-bp, detection for gene Nitab4.5_0002130g0010.1
qNt4-F/ qNt4-R	CCCACCTTATTGTGATCCTGAA<br>AGGCACTGACAAGCCATCCACT		91-bp, detection for gene Nitab4.5_0000274g0040.1

qNt5-F/ qNt5-R	CTCCAAACAGCCTCATCCTC<br>CATGACAGACATGCCACAGC	138-bp, detection for gene Nitab4.5_0002967g0020.1
qNt6-F/ qNt6-R	GCGAAATCTGTGGATAAGGGTG<br>CTGATAGCACAAAGTTGGAGCA	105-bp, detection for gene Nitab4.5_0002824g0090.1
qNt7-F/ qNt7-R	CGGTTTAAGCCATGAGTATGA<br>AATGCTTGCCTTGTGACCTCT	150-bp, detection for gene Nitab4.5_0002239g0020.1
qNt8-F/ qNt8-R	TGAACTGTTTGAGATGGCTGAG<br>TGACCATAAACCACCACTCCTG	147-bp, detection for gene Nitab4.5_0000045g0110.1
qNt9-F/ qNt9-R	GAATAACATTTGAGCCAGAACG<br>TCAAATCCTGGATAATAGGGTG	129-bp, detection for gene Nitab4.5_0002381g0080.1
qNt10-F/ qNt10-R	TATGGGTCGGTGAGAAGAGTTT<br>GGATTTGACTTCATTTGGAGGG	108-bp, detection for gene Nitab4.5_0000382g0070.1

The 5′ end of each primer contains a restriction enzyme site for cloning into the expression plasmids.

References

1. Schmidt, G.W.; Delaney, S.K. Stable internal reference genes for normalization of real-time RT-PCR in tobacco (*Nicotiana tabacum*) during development and abiotic stress. *Mol Genet Genomics*. **2010**, *283*, 233-241.

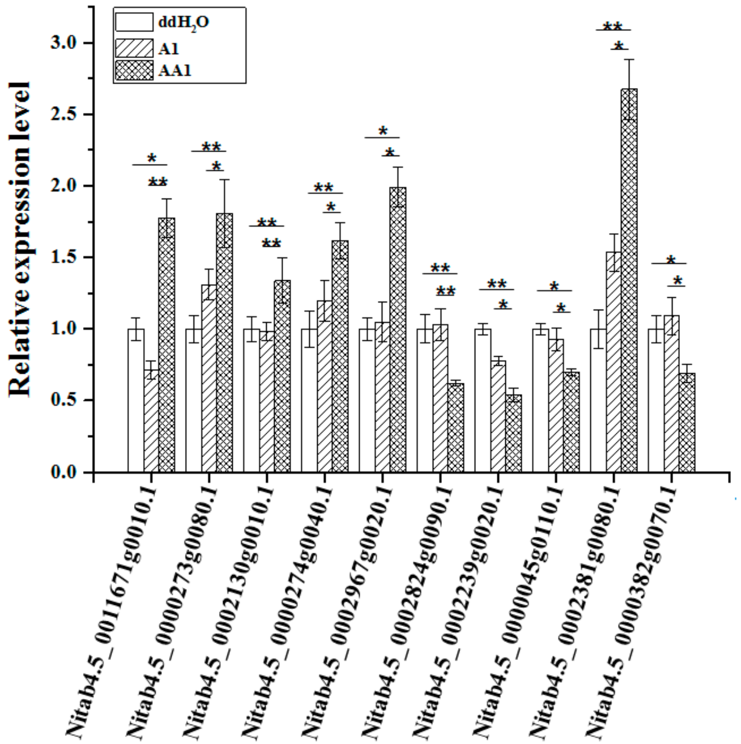
ddH2O
A1
AA1
Relative expression level
0.0
0.5
1.0
1.5
2.0
2.5
3.0
Nitab4.5_0011671g0010.1
Nitab4.5_0000273g0080.1
Nitab4.5_0002130g0010.1
Nitab4.5_0000274g0040.1
Nitab4.5_0002967g0020.1
Nitab4.5_0002824g0090.1
Nitab4.5_0002239g0020.1
Nitab4.5_0000045g0110.1
Nitab4.5_0002381g0080.1
Nitab4.5_0000382g0070.1

Supplementary Figure S1. qRT-PCR analysis of ten genes from 1525 differentially expressed genes in *N. tabacum* roots for different treatments. RNA was extracted from roots collecting from 4-week-old *N. tobacum* NC89 plants at 2 days after inoculation with ddH_2O, 5 mL of a 1 $\times 10^8$ CFU/mL cell suspension of *P. mosselii* A1 or AA1. The blank column represents the sample treated with ddH_2O, the column filled with diagonal rod represents the sample treated with *P. mosselii* A1, and the column filled with grid represents the sample treated with *P. mosselii* AA1. The sequence and annotation of all the tested genes were referred to the database (https://solgenomics.net/organism/Nicotiana_benthamiana/genome). Error bars represented the standard deviation from three independent experiments. Differences were evaluated using Student's t-tests (*P <0.05 and **P < 0.01).

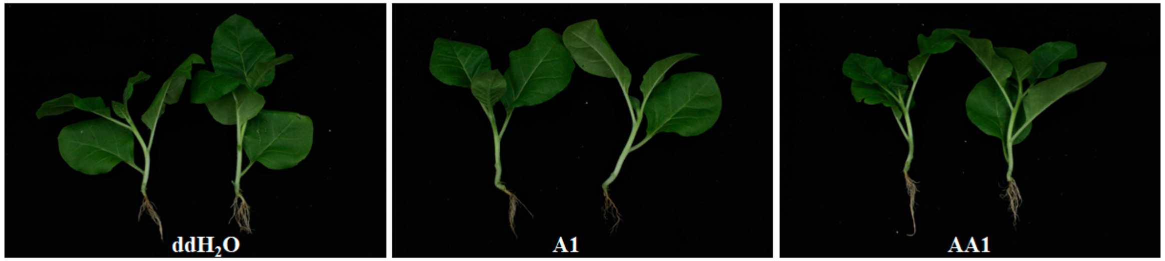

Supplementary Figure S2. The plant growth phenotype among *N. tabacum* by three different treatments. The germinated tobacco seedlings were plant in the soil inoculated with ddH_2O, 5 mL of a 1 $\times 10^8$ CFU/mL cell suspension of *P. mosselii* A1 or AA1. And the phenotype was recorded at 6 weeks after inoculation.

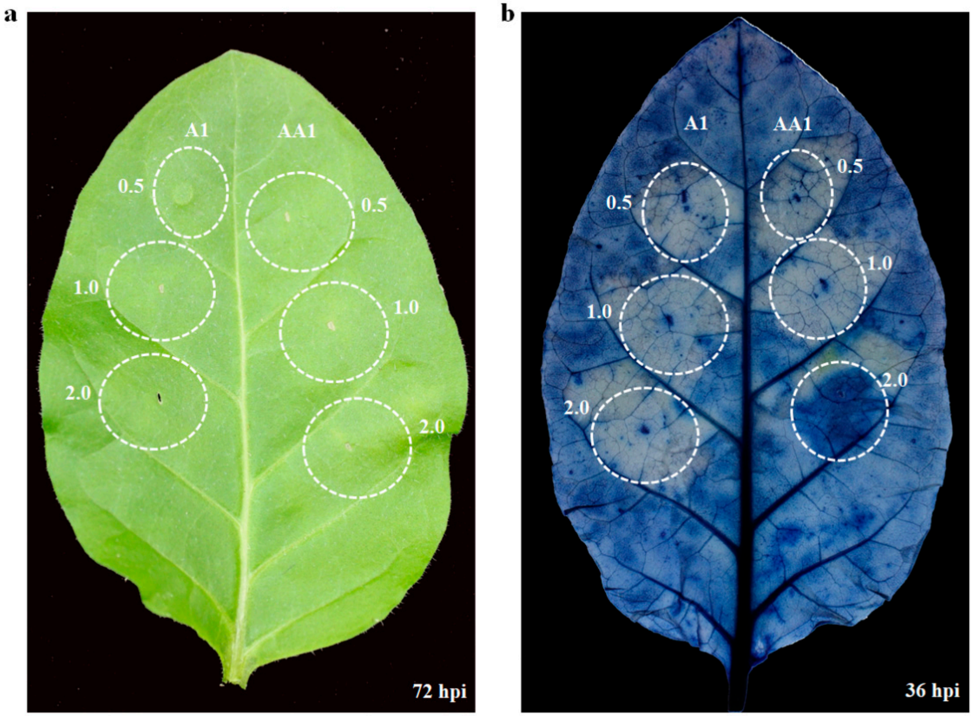

Supplementary Figure S3. Cell death in *N. tabacum* leaves was induced by high concentration of *P. mosselii* AA1: (a) no obvious phenotype in tobacco leaves after inoculation with *P. mosselii* A1 or AA1 strain. The dotted circles indicated the inoculated areas. The different inoculation concentrations of *P. mosselii* A1 or AA1 strain were OD600=0.5, 1.0 and 2.0, respectively. Photographs were taken at 3 days postinfiltraion. (b) detection of plant cell death. Tobacco leaves (36 h postinfiltration) were tested by trypan blue staining. The infiltrated leaves were collected and boiled in a 1:1 mixture containing 96% ethanol and staining solution (10 mL lactic acid, 10 mL phenol, 10 mL glycerol, 10 mL H_2O and 10 mg trypan blue) for approximately 5 min until its green colour has vanished completely visible to the naked eye. The leaves were then detained in detaining solution (dissolve 250 g chloral hydrate into 100 mL H_2O) overnight.
